# A Unique Mode of Failure in the Noncontact Bridging Periprosthetic Plating System

**DOI:** 10.5435/JAAOSGlobal-D-20-00143

**Published:** 2021-02-02

**Authors:** Erin Stockwell, Matthew A. Mormino, Justin C. Siebler

**Affiliations:** From the University of Nebraska Medical Center, Omaha, NE.

## Abstract

Although lateral locking plates are often a preferred and successful fixation construct for the treatment of periprosthetic proximal and distal femur fractures, specific complications and modes of failure have been associated and well-described with their use. We present two cases of implant failure in the Non-Contact Bridge Periprosthetic Plating System (Zimmer Biomet) in which a nonlocked screw fretted through the annular seating of the plate. One case demonstrates failure in the setting of a proximal femur periprosthetic fracture, whereas the other demonstrates failure in the setting of a distal femur periprosthetic fracture. This unique mode of failure has not previously been reported in the literature.

As the rate of hip and knee arthroplasty continues to increase, so have the rates of associated complications, including periprosthetic fractures.^1,2,3,4^ Many periprosthetic fractures of both the proximal and distal femur are successfully managed with the placement of a lateral locking plate.^1,3,5,6,7,8,9^ Several patient-based risk factors and technical errors have been identified as reasons for the failure of these constructs, and examples of implant fracture or screw pull-out have been well documented.^5,9,10,11,12,13^ We have experienced two cases in which a standard screw placed in the diaphyseal portion of the Non-Contact Bridging (NCB) Periprosthetic Plating System (Zimmer Biomet) lost fixation because of fretting through the plate hole. Currently, no previous reports or investigations of this specific complication exist in the literature.

## Case 1

**Figure 1:**
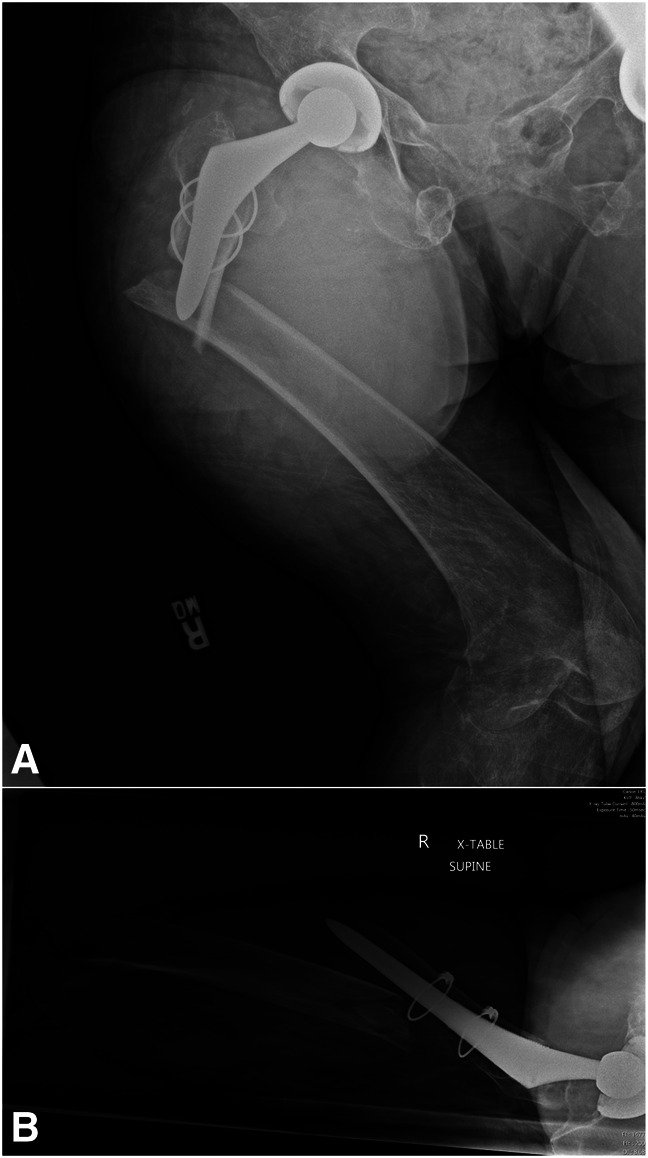
**A**, AP and (**B**) lateral radiographs of the patient's right hip immediately upon presenting to the emergency department demonstrate a displaced and comminuted periprosthetic spiral fracture of the proximal femur. The femoral implant appears to remain well-fixed.

**Figure 2:**
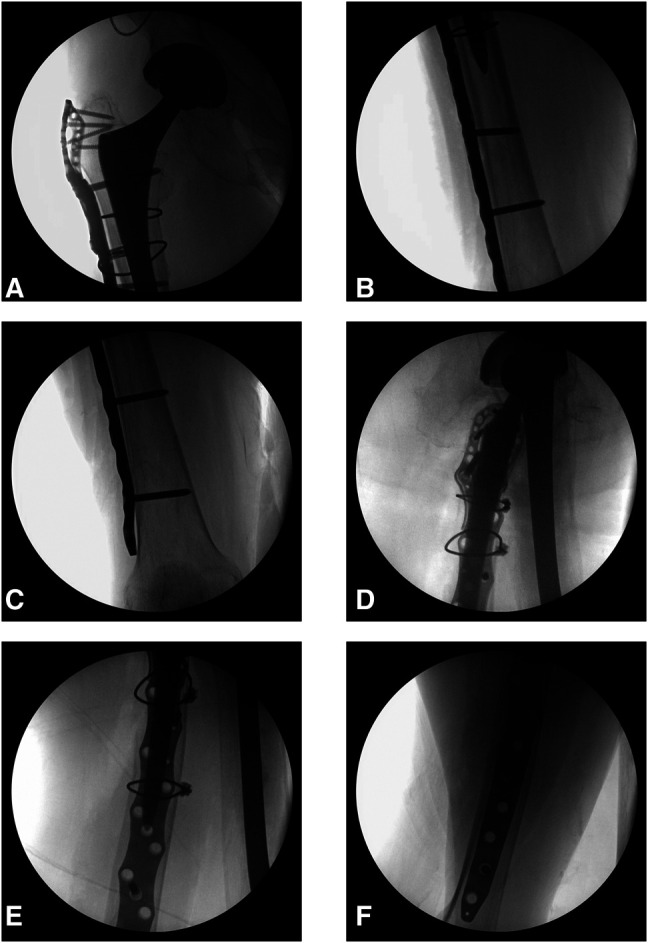
**A**-**C**, AP and (**D**-**F**) lateral intraoperative radiographs of the patient's right femur at the time of open reduction and internal fixation.

An 85-year-old woman with a history of dementia and previously treated osteoporosis presented for the treatment of a right proximal femur Vancouver B1 periprosthetic fracture after sustaining a mechanical ground level fall (Figure 1). She had a remote history of a stroke with associated residual right-sided weakness and used a walker with ambulation at baseline. On admission, she was diagnosed with a urinary tract infection and appropriate antimicrobial therapy was initiated. Blood cultures were negative. She was medically optimized and taken to the operating room the following day for open reduction and internal fixation (Figure 2). A subvastus approach to her femoral shaft was used, and after placing one cerclage wire around the fracture, a titanium alloy NCB Periprosthetic Plate (Zimmer Biomet) with a greater trochanteric attachment was placed. Fixation was obtained via the placement of an additional cerclage wire around the bone and NCB plate, several 3.5 mm and 4.0 mm titanium alloy proximal locking screws, and three 5.0 mm titanium alloy standard screws distal to the fracture. She was discharged on postoperative day 4 back to her assisted living facility. She was allowed to participate in the range of motion as tolerated but was limited to toe-touch weight bearing for 6 weeks. Before discharge, she was evaluated by our endocrinology team who obtained laboratory test results including comprehensive metabolic panel, vitamin D 25 hydroxy, and inorganic phosphate. Several laboratory values were found to be abnormal, and the patient was ultimately started on calcium, vitamin D, and nutritional supplements.

**Figure 3:**
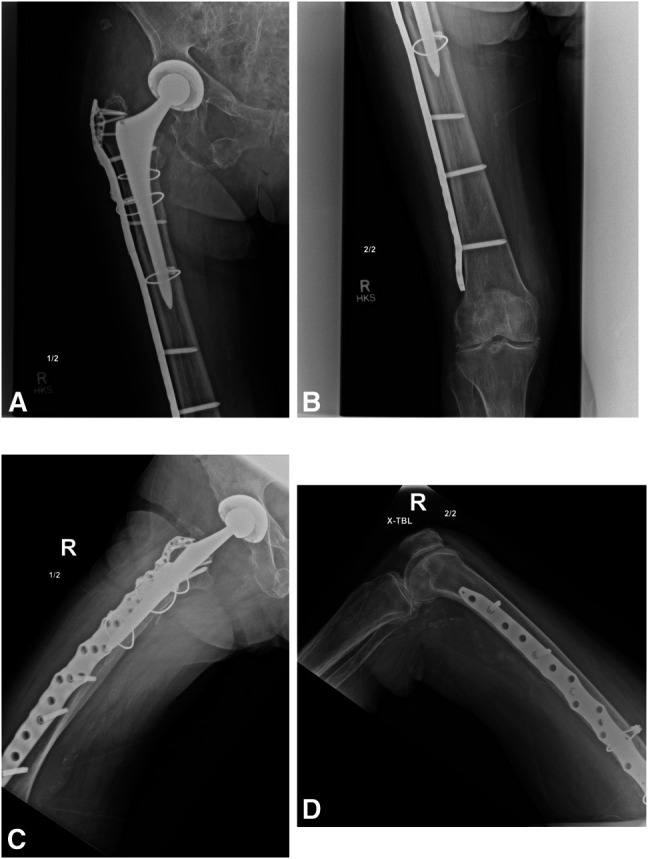
**A** and **B**, AP and (**C** and **D**) lateral radiographs of the right femur obtained 7 weeks postoperatively demonstrate appropriate fracture healing and no evidence of implant failure.

**Figure 4:**
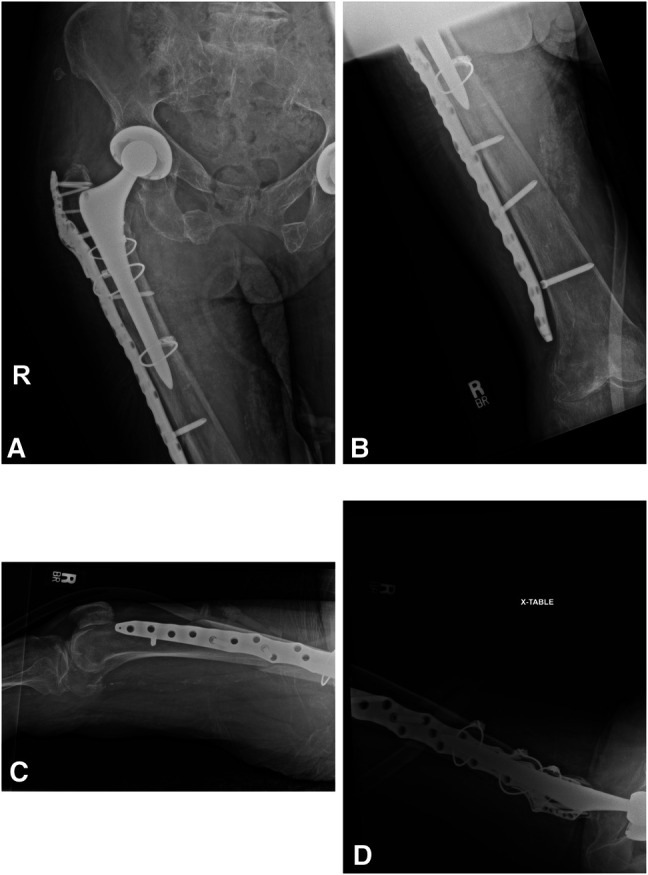
**A** and **B**, AP and (**C** and **D**) lateral radiographs obtained approximately 11 weeks postoperatively at the time of patient re-presentation to the emergency department for evaluation of right lower extremity pain after a fall. There is an acute supracondylar distal femur fracture as well as evidence of the most distal screw having fretted through the plate.

Given the patient's notable commute to our facility, her sutures were removed by her primary care provider and radiographs obtained approximately 7 weeks postoperatively were sent to the operating surgeon for review (Figure 3). At that time, the patient was reported to be doing well. Radiographs demonstrated appropriate fracture healing without evidence of implant failure. Approximately 11 weeks after surgery, she again presented to our emergency department for right lower extremity pain after sustaining an additional mechanical ground level fall. Discussion with family noted that the patient had overall seemed to be recovering well from her previous injury and had recently started painless partial weight bearing activities. Radiographs at that time unfortunately revealed an acute right distal femur fracture and fretting of the distal-most screw through the plate (Figure 4). Standard preoperative laboratory studies were obtained and found to have values within normal limits with the exception of a mild anemia. The patient again underwent open reduction and internal fixation of her acute fracture after medical optimization. She was discharged on postoperative day 6 back to her assisted living facility. The patient was ultimately lost to further follow-up because she entered hospice care on postoperative day 12.

## Case 2

**Figure 5:**
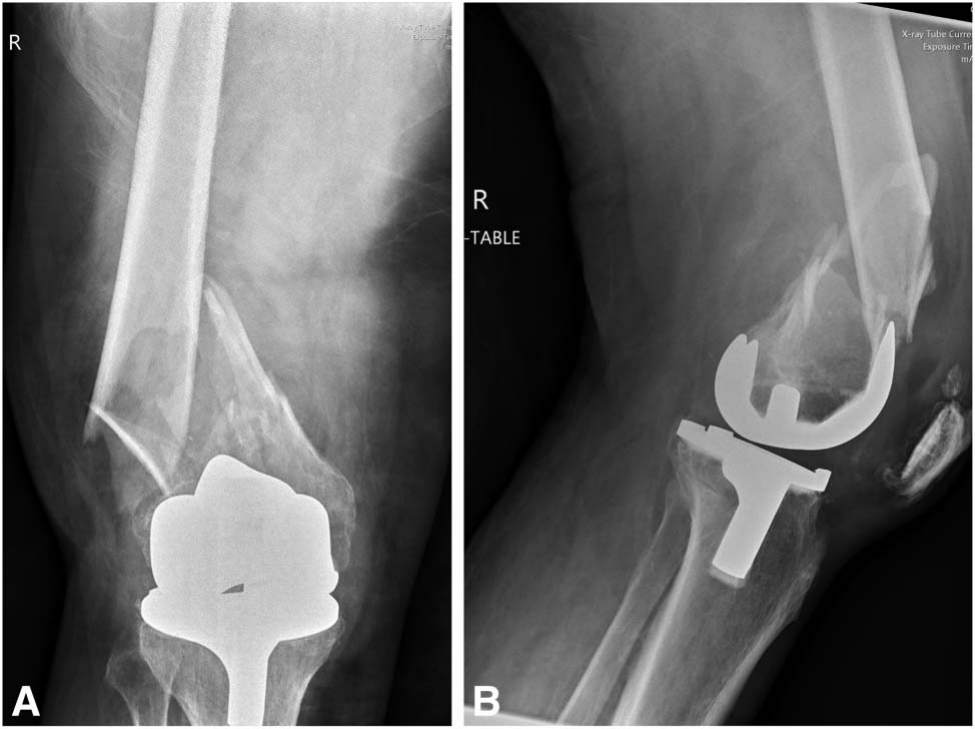
**A**, AP and (**B**) lateral radiographs of the patient's right knee immediately upon presenting to the emergency department demonstrate a comminuted and displaced periprosthetic supracondylar distal femur fracture.

**Figure 6:**
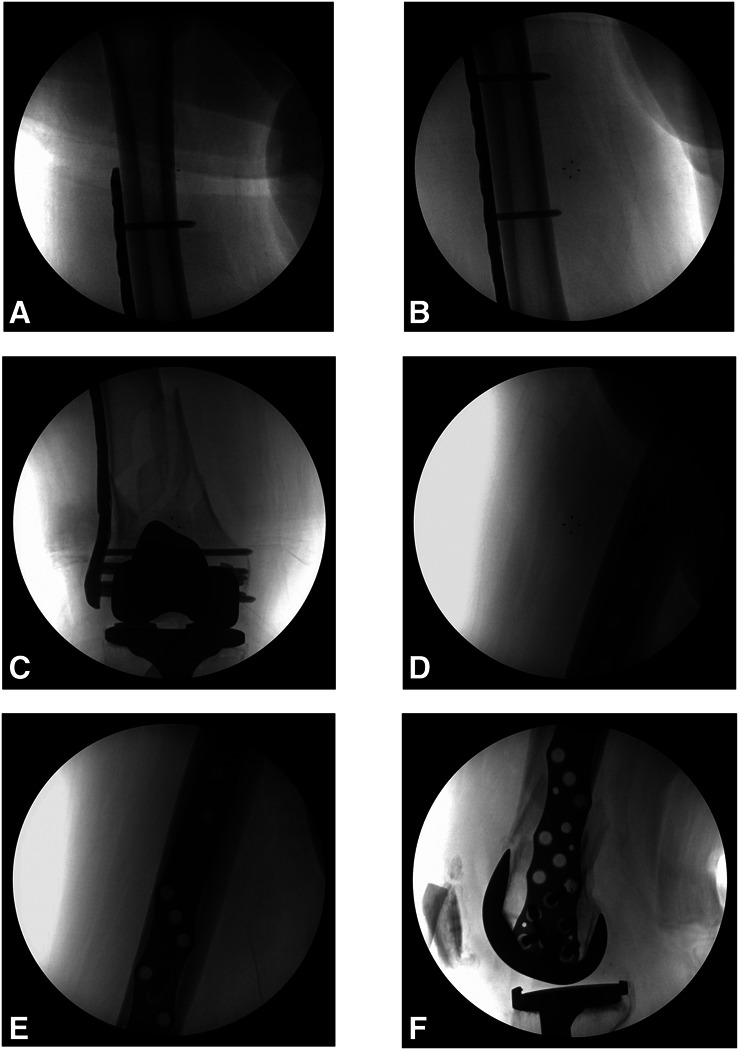
**A**-**C**, AP and (**D**-**F**) lateral intraoperative radiographs of the patient's right femur at the time of open reduction and internal fixation.

A 73-year-old man with multiple medical comorbidities including multiple myeloma after chemotherapy, diabetes, and coronary artery disease presented for the treatment of a comminuted right periprosthetic supracondylar distal femur fracture after sustaining a mechanical ground level fall (Figure 5). Before this injury, he was able to ambulate two blocks without difficulty but did use a stair lift at home secondary to chronic left lower extremity weakness, which he attributed to his previous multiple myeloma treatment. He was medically optimized and taken to the operating room the following day for open reduction and internal fixation (Figure 6). After achieving adequate reduction of the fracture through indirect reduction techniques, a limited lateral approach to the distal femur was performed and the proximal end of a titanium alloy NCB Periprosthetic Plate (Zimmer Biomet) was placed submuscularly. Three titanium alloy 5.0 mm standard screws were placed in a percutaneous manner proximal to the fracture and multiple titanium alloy 5.0 mm locked screws were placed in the distal segment. Postoperatively, he was placed in a knee immobilizer to be worn at all times for 3 weeks, at which time the range of motion exercises were initiated. He was limited to toe-touch weight bearing for 8 weeks. Before discharge, he was evaluated by our endocrinology team who obtained laboratory test results including comprehensive metabolic panel, vitamin D 25 hydroxy, and inorganic phosphate. Several of these laboratory values were found to be abnormal, and he was ultimately started on calcium and vitamin D supplements.

**Figure 7:**
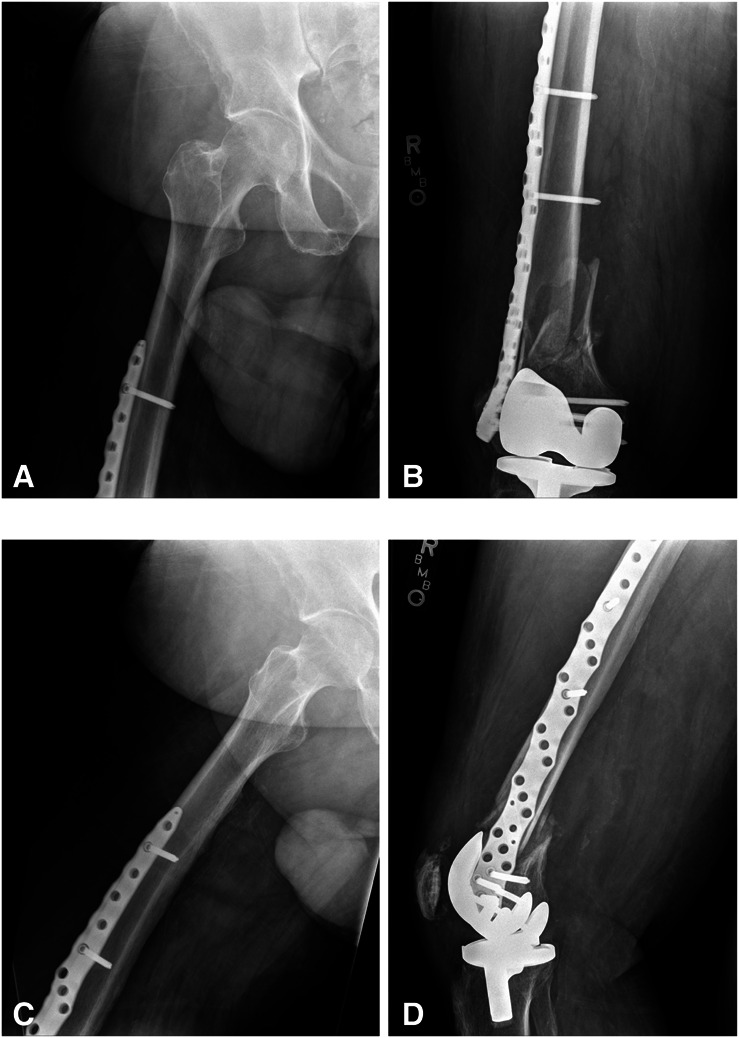
**A** and **B**, AP and (**C** and **D**) lateral radiographs of the right femur obtained 8 weeks postoperatively demonstrate early callous formation without evidence of implant failure.

The patient was discharged to a skilled nursing facility on postoperative day 5. At his 8-week postoperative appointment, new radiographs were obtained (Figure 7). Adequate callous formation was noted, and no concerns for complications were noted. At that time, he did have notable quadriceps atrophy but denied pain with passive or active range of motion. He was advanced to weight bearing as tolerated and given home quadriceps strengthening exercises.

**Figure 8:**
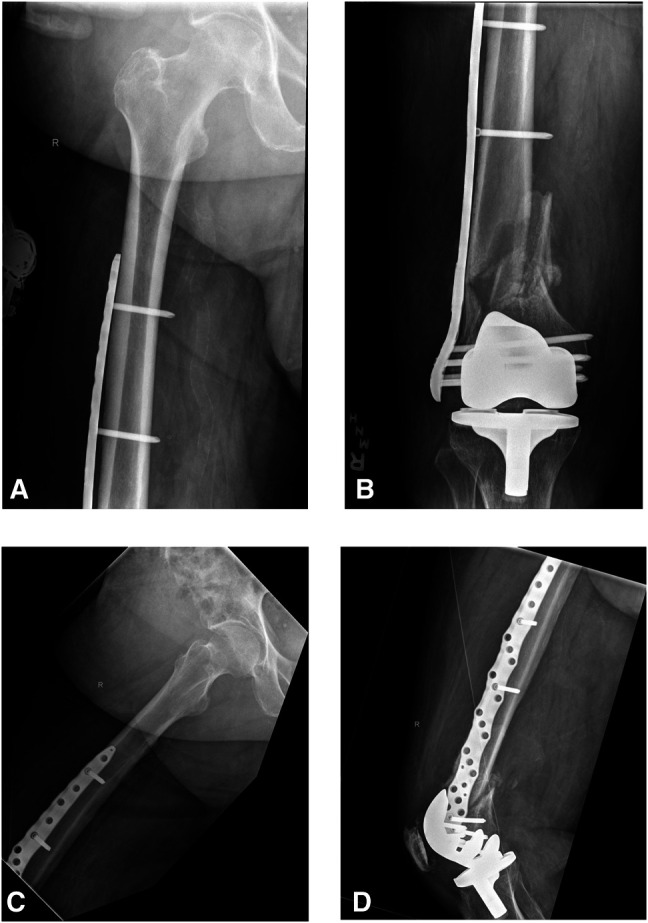
**A** and **B**, AP and (**C** and **D**) lateral radiographs of the right femur obtained 14 weeks postoperatively demonstrate slight varus malalignment of the fracture with fretting of the most distal screw proximal to the fracture line through the plate.

At his 14-week postoperative appointment, it was noted that the patient had not been compliant with his home strengthening exercises, and he reported 75% weight bearing with continued use of a walker for all ambulation. He endorsed stiffness in his right knee and intermittent mild pain described as muscle soreness in his distal thigh. Radiographs were obtained, which demonstrated slight varus malalignment of the fracture and fretting of the distal-most screw placed proximal to the fracture site (Figure 8). He was referred to physical therapy but was unfortunately lost to further follow-up.

## Discussion

Periprosthetic femur fractures are complex orthopaedic injuries that often occur in patients with multiple medical comorbidities and poor bone quality.^1,5,10,14^ In the setting of a periprosthetic fracture around a stable prosthetic implant, these injuries have historically been treated with fixed-angle devices, blade plates, intramedullary nails, or lateral locking plates.^3,5,8,9,14^ Lateral locking plates have quickly become the preferred treatment option for many periprosthetic femur fractures because of their excellent stability and ability to be placed in a minimally invasive manner.^5-7,10,13,14^

Despite being the treatment of choice in certain fracture patterns, various characteristics have been identified to play a role in the development of clinical complications and implant failure.^5,11-13,15^ In a large retrospective review of 335 distal femur fractures, Ricci et al^12^ found smoking, open fracture, and shorter overall plate length to be independent risk factors for experiencing implant failure. Neither of our patients possessed these specific risk factors, although both were noted by the endocrinology team to be osteoporotic, and the patient in case 1 was found to be malnourished. Review of final intraoperative imaging confirms appropriate fracture alignment and rigidity of both fixation constructs. Although some concern may be had regarding patient compliance with toe-touch weight bearing instructions, all reported information from the patient and their caregivers indicated patient compliance with restrictions. In summary, an obvious etiology of implant failure in these cases is not clearly identified.

The most common complications associated with the use of lateral locking plates in the treatment of femur fractures include nonunion, implant fracture, and wound infection.^6,7,12^ The rate of delayed union and nonunion associated with the use of lateral locking plates ranges from 0% to 32% within the literature.^5-7,12^ Less common but documented complications include prominent screws requiring hardware removal, symptomatic heterotopic ossificans, and persistent pain.^3,7,14^ There are numerous clinical examples of plate and screw fracture or screw pull-out, but no literature exists demonstrating the unique mode of failure exhibited in our clinical cases in which a standard screw remained within the bone and subsequently fretted through the annular seating of the plate. It is possible that the application of these screws in a locked fashion would have prevented this mode of failure. Additional biomechanical testing is required to elucidate the cause of this mode of failure and its prevention.

Several methods are available to surgeons to manipulate the rigidity of a fixation construct, including the placement of standard screws in the diaphyseal portion of the plate.^3,14,16^ With the knowledge of this failure mode with this particular plate, it may be advantageous to choose a different method to manipulate fracture construct rigidity. Several studies have demonstrated increased complications related to fracture healing in overly stiff fixation constructs.^5-7,10,11,13^ Based on this knowledge paired with our experiences with this specific implant, we consider placing a locked screw adjacent to the fracture within the femoral shaft while placing standard screws for the remainder of the fixation within the shaft to avoid making the construct too stiff. Surgeons must not only be aware of the available treatment options and the associated complications but also be diligent in critically evaluating the presenting fracture pattern and associated clinical scenario when making treatment decisions.
